# A Roadside Precision Monocular Measurement Technology for Vehicle-to-Everything (V2X)

**DOI:** 10.3390/s24175730

**Published:** 2024-09-03

**Authors:** Peng Sun, Xingyu Qi, Ruofei Zhong

**Affiliations:** College of Resource Environment and Tourism, Capital Normal University, Beijing 100048, China; 1213602010@cnu.edu.cn (P.S.); 2220902187@cnu.edu.cn (X.Q.)

**Keywords:** YOLOv5x, object recognition, single-image distance measurement, traffic safety, advisory system, pedestrian safety

## Abstract

Within the context of smart transportation and new infrastructure, Vehicle-to-Everything (V2X) communication has entered a new stage, introducing the concept of holographic intersection. This concept requires roadside sensors to achieve collaborative perception, collaborative decision-making, and control. To meet the high-level requirements of V2X, it is essential to obtain precise, rapid, and accurate roadside information data. This study proposes an automated vehicle distance detection and warning scheme based on camera video streams. It utilizes edge computing units for intelligent processing and employs neural network models for object recognition. Distance estimation is performed based on the principle of similar triangles, providing safety recommendations. Experimental validation shows that this scheme can achieve centimeter-level distance detection accuracy, enhancing traffic safety. This approach has the potential to become a crucial tool in the field of traffic safety, providing intersection traffic target information for intelligent connected vehicles (ICVs) and autonomous vehicles, thereby enabling V2X driving at holographic intersections.

## 1. Introduction

In recent years, the rapid development of intelligent transportation systems and new infrastructure has ushered in a new phase for vehicle–road collaboration [1]. In this context, the collaborative perception and decision-making capabilities of roadside sensors have become increasingly important. This raises higher demands on the precision, latency, and accuracy of roadside information data. Holographic intersections, as a key focus in the upgrade of urban intelligent transportation infrastructure, require refined perception data and real-time detection capabilities to address the complexities of traffic environments. Traditional intersection cameras often struggle to meet the high-precision distance measurement requirements, making an upgrade of roadside perception capabilities imperative [2,3,4].

To address this issue, this study proposes an automated vehicle distance detection and warning system based on roadside camera video streams. This system transmits camera data to an edge computing unit, which performs intelligent processing to output high-precision target-level data [5]. The intelligent processing employs a target recognition method based on the YOLOv5x neural network model and estimates distances using single-image measurement principles [6] based on similar triangles, providing safety recommendations based on distance estimates to reduce traffic accident rates and ensure pedestrian safety [7].

Compared to traditional multi-sensor fusion methods [8], this study simplifies the system by using a single camera for distance measurement, significantly reducing system complexity and cost. For target recognition, we combined the Canny algorithm for edge detection, which enables the system to more accurately identify and detect vehicle and pedestrian edge features, thereby enhancing target recognition performance. Experimental data validate that this strategy achieves centimeter-level distance detection accuracy, contributing to a reduction in traffic accidents and improved pedestrian safety.

The contributions of this paper can be summarized as follows:

1. A processing framework combining target recognition, distance measurement, and vehicular networking technologies is proposed, providing a new solution for traffic safety that effectively enhances traffic accident prevention and pedestrian safety.
2. The target recognition output is improved by integrating the Canny algorithm for edge extraction, allowing for more accurate extraction of target edge features and thus enhancing target recognition performance. This improvement enables the system to better adapt to complex traffic environments and increases recognition accuracy and stability.
3. Experimental data validate that the proposed processing framework achieves significant accuracy in distance detection. This demonstrates the superiority of the framework in accurately measuring distances between vehicles and pedestrians. The results provide a feasible solution for traffic safety and robust support for traffic accident prevention and pedestrian safety in practical applications.

## 2. Related Work

In the past few decades, traffic accidents have become a significant factor leading to casualties and property loss. Therefore, research on how to prevent traffic accidents and ensure pedestrian safety has been a focus in the transportation field. Previous studies have focused on the relationship between vehicles. Liqin Huang et al. [9] introduced a robust vehicle distance estimation method based on monocular vision, utilizing principles of object detection, segmentation, and geometric analysis for distance estimation. Ahmed Ali et al. [10] proposed a real-time vehicle distance estimation method based on single-view geometry, utilizing geometric features of road lane markings and combining lane and vehicle detection modules to estimate the intersection ratio of the horizon and calculate the distance between vehicles and the road. Karthika K. et al. [11] presented a method for preceding vehicle distance estimation based on monocular vision cameras and artificial neural networks, utilizing a YOLO object detector for vehicle detection and extracting input and output attributes from detected vehicle bounding boxes to estimate preceding vehicle distances. Deng-Yuan Huang et al. [12] described a driving assistance system based on a single-lens video camera, providing drivers with assistance in vehicle detection and distance estimation on urban and suburban roads to improve driving safety. Giseok Kim et al. [13] introduced a vision-based vehicle detection and inter-vehicle distance estimation algorithm for driving assistance systems, which combined multiple vehicle and Haar-like features as well as edge features to accurately detect vehicles and estimate distances between them in real-time scenarios. J. Han et al. [14] presented a method for vehicle distance estimation using a monocular camera, utilizing detected lane information and a pinhole camera model to calculate the position, velocity, acceleration, and time-to-collision (TTC) of target vehicles. The above-mentioned articles have developed various methods and technologies to achieve distance estimation between vehicles in monocular vision, without considering the safety environment of surrounding pedestrians.

Safety recommendations based on distance estimation are a key step in improving traffic safety. Tabitha S. Combs et al. [15] proposed that equipping vehicles with VLC, LiDAR, and radar devices could significantly reduce the likelihood of pedestrian fatalities, based on pedestrian fatality data from various states and the District of Columbia in the United States in 2015. Piotr Olszewski et al. [16] conducted research on pedestrian–vehicle encounters in Warsaw and Wroclaw, based on MOBIS project data, to develop alternative safety indicators for pedestrian–vehicle encounters using parameters such as speed profiles of pedestrians and vehicles, minimum distances between participants, and deceleration during braking. They also proposed encounter classifications based on pedestrian–vehicle interaction characteristics and evaluated solutions to enhance pedestrian safety at crosswalks. Alexander Ganichev et al. [17] aimed to address conflicts between vehicles and pedestrians and proposed a systemic approach to improve traffic safety. They considered evaluating conflicts between vehicles and pedestrians within the (P-V-R-TE) system as a promising method. This approach, based on objective analysis and assessment, aimed to reduce conflicts between vehicles and pedestrians through the systematic analysis and improvement of traffic management measures, thereby providing more reliable decision-making grounds to enhance traffic safety. Salvatore Cafiso et al. [18] presented a method for measuring traffic conflicts based on the Pedestrian Risk Index (PRI) to assess the impact of new traffic calming devices on pedestrian crossing safety performance. Je-Seok Ham et al. [19] proposed the CIPF model, a network for predicting pedestrian crossing intentions, aimed at improving the safety of autonomous vehicles with respect to pedestrians. This model, by integrating multiple input features of pedestrians and vehicles and utilizing recurrent neural networks and attention mechanisms, accurately predicted whether pedestrians would cross the road. These papers provide a series of safety recommendations such as deceleration and maintaining safe distances, based on rough estimations of pedestrian safety environments, combined with traffic rules and safety standards, to reduce the occurrence of traffic accidents and ensure pedestrian safety.

In summary, previous research has primarily focused on preventing vehicle-to-vehicle traffic accidents, target recognition algorithms, distance estimation, and safety recommendations. There has been no relevant research accurately measuring the distance between pedestrians and vehicles. Therefore, building upon these studies, this research proposes a target recognition method based on neural network models, integrating edge extraction using the Canny algorithm, utilizing the principle of similar triangles for distance estimation from a single image, and providing safety recommendations based on distance estimation. The aim is to reduce the occurrence of traffic accidents and ensure pedestrian safety.

## 3. Methodology

### 3.1. System Architecture

**Figure 1:**
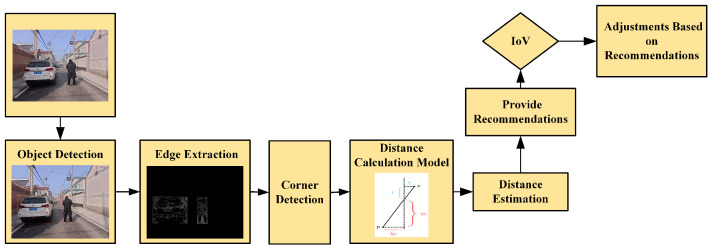
System architecture diagram.

The system is divided into four components: target extraction, edge extraction, distance estimation, and safety recommendations (as shown in Figure 1). The system processes real-time images captured by the camera, performing preprocessing to enhance the accuracy and effectiveness of target recognition and edge extraction. A neural network model, such as YOLOv5x, is used for target recognition to detect objects such as vehicles and pedestrians in the images. The Canny algorithm is applied to extract edges from the images within the detection boxes to enhance the edge information of the targets. Based on the edge information features, distance estimation is performed using the single-image measurement principle of similar triangles. Safety recommendations are generated based on the distance estimates.

### 3.2. Target Recognition

In our study, the YOLOv5x model [20] was used and trained with the COCO dataset due to several notable advantages. Firstly, the YOLOv5x model demonstrates exceptional real-time performance while ensuring high accuracy, which is crucial for applications requiring rapid response to traffic intersection video streams. Secondly, the model exhibits outstanding generalization capability across various datasets, effectively handling diverse object detection tasks. Moreover, the training process for YOLOv5x is straightforward and intuitive, facilitating fine-tuning and optimization. Additionally, the model’s relatively small size and high computational efficiency make it suitable for deployment on resource-constrained devices, which is essential for its application in roadside cameras.

The COCO dataset, which stands for “Common Objects in Context”, is a widely used computer vision dataset published by Microsoft Research. It is primarily used for tasks such as object detection, image segmentation, and image captioning. The COCO dataset includes a variety of everyday objects across 80 categories, including people, animals, vehicles, and household items. It contains over 200,000 images, with approximately 330,000 images annotated with over 1.5 million object instances. COCO not only focuses on the objects themselves but also on the contextual relationships between objects, providing a more challenging learning task for models. Its rich annotation information and diverse task applications make it an ideal choice for training and evaluating tasks such as object detection, image segmentation, keypoint detection, and image captioning.

Therefore, based on these advantages, we selected YOLOv5x as our object detection model and used the COCO dataset for training. This choice enhanced our research efficiency and ensured that our results would be highly accurate and practical.

**Figure 2:**
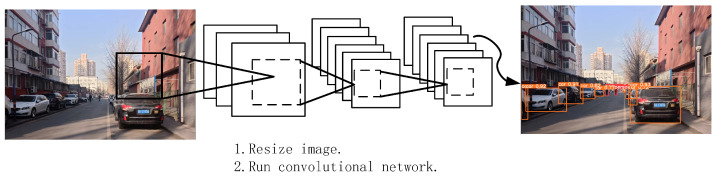
Network prediction process.

The prediction process of YOLOv5 (as shown in Figure 2) involves the following steps: Initially, the input image is resized to the dimensions required by the model. Subsequently, the image is fed into the convolutional neural network, which extracts image features and predicts multiple bounding boxes, confidence scores, and class probabilities. Then, YOLOv5 employs non-maximum suppression (NMS) to eliminate redundant bounding boxes, retaining only those with the highest confidence scores. Finally, the model outputs detection results that include bounding boxes, confidence scores, and class labels. These results can be further processed or displayed for various applications.

### 3.3. Edge Extraction

**Figure 3:**
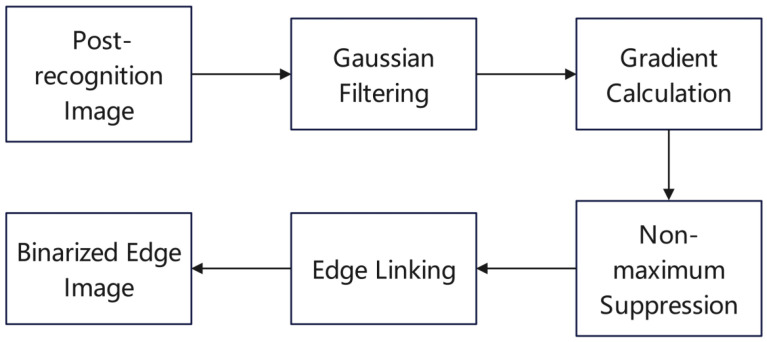
Canny algorithm flowchart.

The Canny algorithm is a classical edge detection technique that precisely extracts edge information from an image (as shown in Figure 3). Initially, Gaussian smoothing is applied to reduce the impact of noise. Subsequently, the gradient magnitude and direction of the image are calculated, typically using the Sobel operator to obtain gradient information for each pixel. Then, non-maximum suppression is performed to retain only the most significant edges and refine these edges. Following this, double threshold processing classifies the gradient magnitudes into strong edges, weak edges, and non-edges. Strong edges are directly marked as edges, while weak edges are assessed to determine whether they are connected to strong edges. Finally, edge refinement is carried out to remove redundant pixels, making the edges thinner and more continuous.

In traffic safety warning systems, the Canny algorithm not only complements the object detection results of YOLOv5 but also significantly enhances the system’s performance. By integrating the Canny algorithm, the system can accurately extract the contours of cars and pedestrians in complex traffic environments. This helps to eliminate the impact of surrounding objects on distance measurement and improves the accuracy and robustness of detection. Innovatively combining the Canny algorithm with YOLOv5 can achieve more efficient edge detection and target recognition, enabling the system to better cope with complex real-world application scenarios. Such integration contributes to the development of smarter and more reliable traffic safety warning systems, providing drivers with more accurate distance measurements and more reliable safety advice.

### 3.4. Distance Estimation

**Figure 4:**
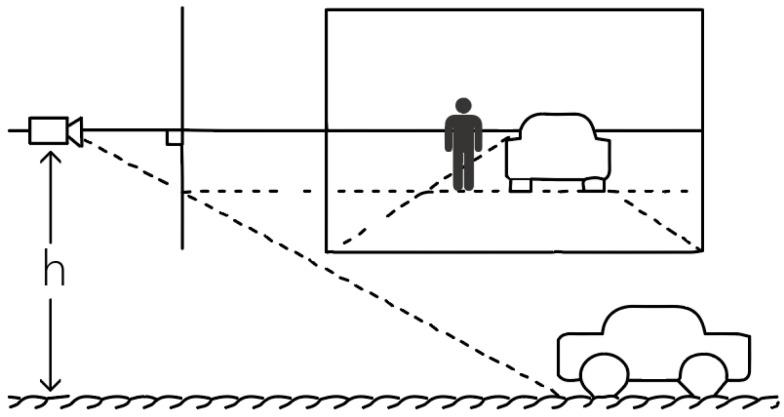
Schematic diagram of human and vehicle status in a single image.

This study employed Zhang’s method for camera calibration, processing multiple images of a chessboard pattern taken from different angles. The chessboard corners were extracted from the images and combined with the known physical dimensions of the chessboard, and a nonlinear optimization algorithm was used to estimate the camera’s intrinsic parameters, such as focal length, principal point coordinates, and distortion coefficients. These intrinsic parameters were not only utilized to correct image distortions but also provided precise geometric relationships for subsequent distance measurements, ensuring the accurate estimation of object positions in the Earth-centered coordinate system. The calibrated camera was fixed at a height of 3 m on a streetlamp, aligned parallel to the ground, as shown in Figure 4. The camera’s orientation was aligned with the road axis to ensure that the captured images accurately reflected real-world distances.

Since the image captured by the camera was saved as two-dimensional data of pixel points, the coordinates of the object in the image were represented as (x,y), and the object was located in the Earth’s spatial coordinate system under the camera’s view angle, with its coordinates in the Earth’s spatial coordinate system represented as $\left(X_{C},Y_{C},Z_{C}\right)$, where $\left(X_{0},Y_{0},Z_{0}\right)$ are the spatial coordinates of the photographic center (camera position). From this, the collinearity equation was derived.

(1) $$x=-f\frac{r_{11}\left(X_{C}-X_{0}\right)+r_{12}\left(Y_{C}-Y_{0}\right)+r_{13}\left(Z_{C}-Z_{0}\right)}{r_{31}\left(X_{C}-X_{0}\right)+r_{32}\left(Y_{C}-Y_{0}\right)+r_{33}\left(Z_{C}-Z_{0}\right)}$$

(2) $$y=-f\frac{r_{21}\left(X_{C}-X_{0}\right)+r_{22}\left(Y_{C}-Y_{0}\right)+r_{23}\left(Z_{C}-Z_{0}\right)}{r_{31}\left(X_{C}-X_{0}\right)+r_{32}\left(Y_{C}-Y_{0}\right)+r_{33}\left(Z_{C}-Z_{0}\right)}$$

where $r_{ij}$ is the element of the rotation matrix *R*, which describes the rotational relationship between the Earth coordinate system and the camera coordinate system. *f* is the camera’s focal length. Through these collinearity equations, the object points in the Earth’s spatial coordinate system can be mapped onto the two-dimensional pixel coordinates in the image.

**Figure 5:**
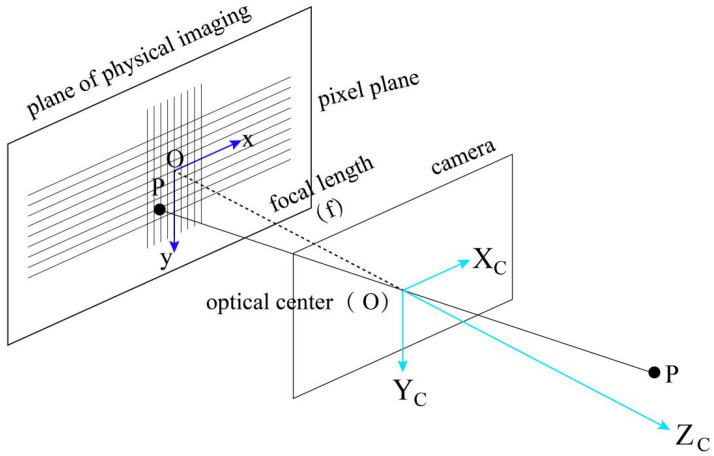
Coordinate Systems in Pinhole Imaging Model.

**Figure 6:**
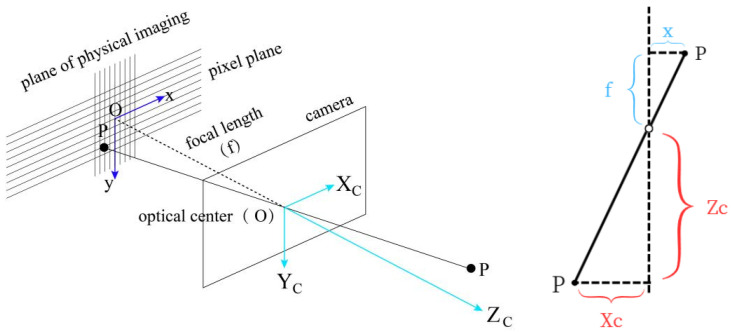
Similar Triangles in Pinhole Imaging.

Simplifying the formula, the transformation between the camera coordinate system and the image coordinate system can be based on the pinhole camera model. This process results in the loss of depth information of the spatial points. By analyzing the geometric relationship between the spatial points and the corresponding image points as shown in Figure 5, we can identify similar triangles in the imaging geometric relationship as depicted in Figure 6. Therefore, there is a coordinate transformation as shown in Equation (3), where *f* is the camera’s focal length.

(3) $$\frac{Z_{C}}{f}=\frac{X_{C}}{x}=\frac{Y_{C}}{y}$$

**Figure 7:**
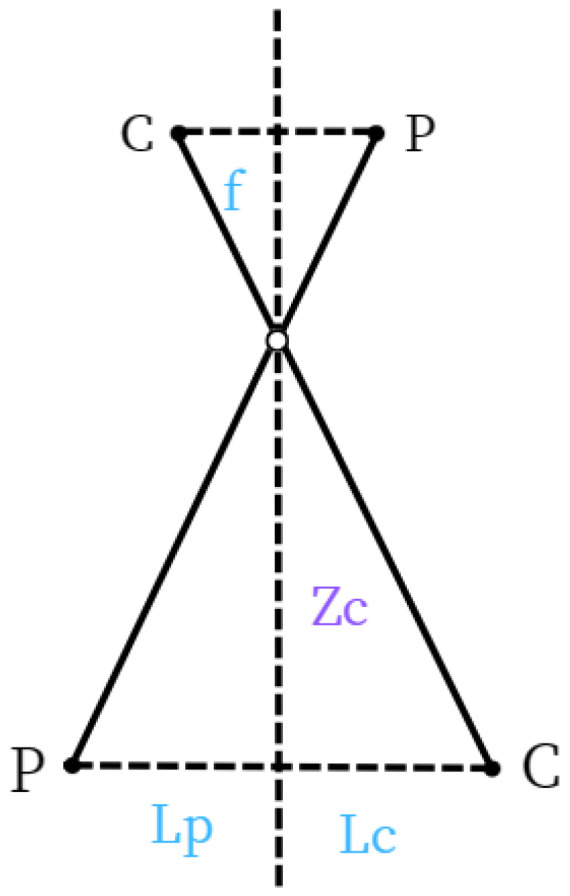
Overhead Distance Relationship Diagram.

Since the unit of the pixel coordinate system is pixels and the unit of the image coordinate system is millimeters, it is necessary to perform a conversion between the image coordinate system and the pixel coordinate system. As this study focused on a pedestrian safety warning system, the camera was positioned at the street edge, two meters above the ground, capturing images vertically. The shooting effect is shown in Figure 4. Therefore, a method was proposed to calculate the pixel ratio in this plane based on the camera’s height and to calculate the actual distance based on the pixel distance, as shown in Figure 7 (where $L_{p}$ represents the distance from the person to the camera’s central axis, and $L_{c}$ represents the distance from the car to the camera’s central axis).

Let $h_{x}$ be the pixel distance from the image center to the ground, and $L_{x}$ be the pixel distance between two points in the same plane as the selected $h_{x}$. *h* stands for the actual height of the camera. Let *d* be the pixel ratio in this plane. From this, we can obtain:

(4) $$\frac{h}{h_{x}}=\frac{L}{L_{x}}=d$$

and calculate *L*:

(5) $$L=\frac{h}{h_{x}}\times L_{x}$$

Then, we can compare *L* with a threshold value and propose relevant safety recommendations.

### 3.5. Communication Protocol between Roadside Units (RSUs) and Vehicles

**Figure 8:**
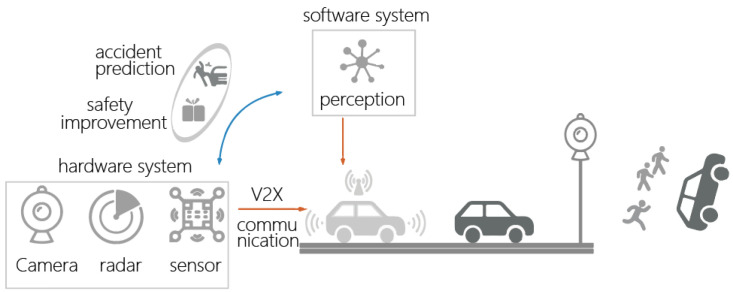
Vehicle-to-Everything (V2X) Vehicle-Road Collaboration System.

To achieve real-time warnings, we implemented a vehicular network system based on the integration of perception, communication, and computation, as illustrated in Figure 8. This system incorporates onboard units (OBUs) and IEEE 802.11p wireless communication technology, aiming to reduce traffic accidents by monitoring the distance between vehicles and pedestrians in real time and providing timely safety alerts.

1. System Architecture

**Figure 9:**
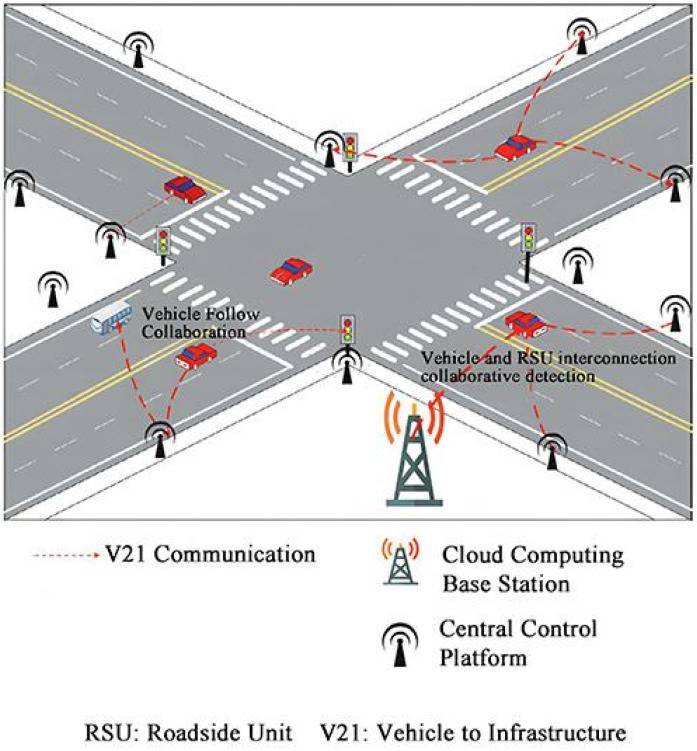
Hardware deployment diagram of vehicular network communication.

The system consists of Roadside Units (RSUs), onboard units (OBUs), and a Central Control Platform, as shown in Figure 9. The RSUs are installed at fixed locations along the road and are responsible for wireless communication with passing vehicles. Each vehicle is equipped with an OBU, which receives instructions from the RSU and transmits vehicle status information back. The Central Control Platform aggregates data from all RSUs and OBUs and performs global analysis and decision-making to optimize traffic management and safety.

2. Communication Protocol Design

To achieve efficient communication between vehicles and RSUs, this study employed the IEEE 802.11p communication protocol [21]. IEEE 802.11p is a wireless communication standard specifically designed for vehicular networks, providing low-latency and reliable communication in high-speed mobility environments. The protocol operates in the 5.9 GHz frequency band, enabling stable communication even when vehicles are traveling at high speeds.

**Figure 10:**
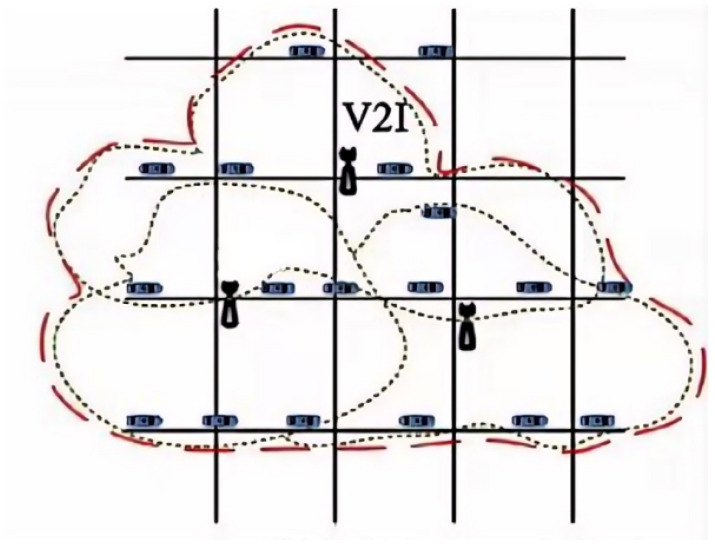
Diagram of Multiple RSU Coverage Areas.

The communication process between RSUs and OBUs in the system was as follows:

(1) Data broadcasting: the RSU continuously broadcasts traffic environment information, including road conditions, pedestrian positions, and dynamic information of other vehicles.
(2) Response mechanism: the onboard Unit (OBU) receives the broadcast information from the RSU and analyzes it in conjunction with the vehicle’s current status (such as speed and position).
(3) Priority management: within the communication protocol, safety warning messages are assigned the highest priority to ensure that these alerts are transmitted quickly and reliably between the OBU and RSU, taking precedence over other non-urgent data.
(4) Vehicle handover: when a vehicle moves from the coverage area of one RSU to another, the system automatically performs seamless handover of vehicle information, ensuring continuity and consistency of warning messages throughout the entire journey, as illustrated in Figure 10.

3. Vehicle Identification and Tracking

Each vehicle’s onboard unit (OBU) is equipped with a unique identifier, such as a license plate number or electronic tag, allowing the system to accurately identify and track specific vehicles. In practical applications, the RSU can establish an independent communication link with the OBU using these identifiers, ensuring that warning messages are accurately delivered to the corresponding vehicle, thereby preventing information confusion or transmission delays.

## 4. Experiment

Based on the algorithm framework developed in this paper, we devised a pedestrian safety warning system to enhance the safety of pedestrians. The system captured real-time video streams through a camera with 1080p resolution and a frame rate of 30 fps and used the NVIDIA Jetson Xavier NX edge computing unit for data processing. The Jetson Xavier NX is equipped with a 6-core ARM CPU, a 384-core Volta GPU, 8 GB of memory, and 512 GB of SSD storage, offering powerful computing capabilities that enable real-time operation of the YOLOv5x object recognition model and high-precision distance estimation based on single image measurement and the principle of similar triangles.

This ensured that the system could accurately detect pedestrians and vehicles and provide corresponding safety warnings based on their location and distance. By processing and analyzing video data in real time, the system could issue timely alerts to remind drivers and pedestrians of potential safety risks, effectively reducing the occurrence of collision accidents and enhancing pedestrian safety.

The system obtained real-time video streams from the camera and processed each frame with the algorithm. A static scene from one of the frames was selected for the actual distance measurement to compare with the algorithm, as shown in Figure 10.

**Figure 11:**
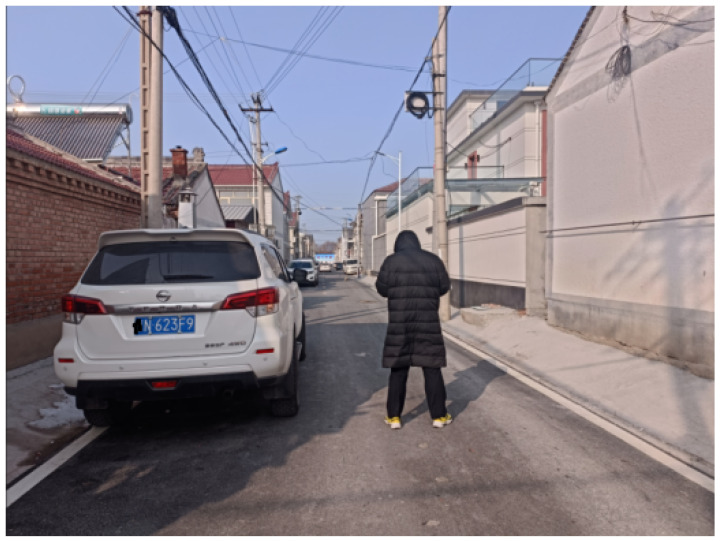
One Frame Image from the Video Stream.

The trained YOLOv5x model was applied to process each frame, detecting and recognizing pedestrians and vehicle targets within it. This enabled us to accurately locate and label the positions of pedestrians and vehicles in the image, as shown in Figure 11.

**Figure 12:**
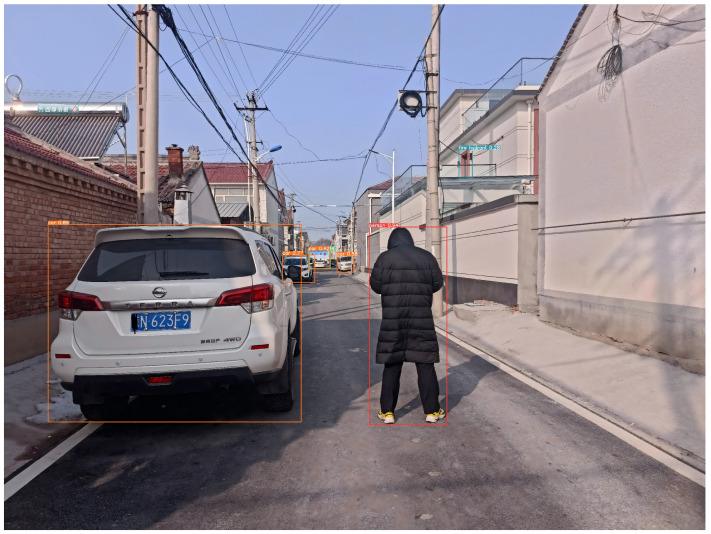
Recognition Results Image.

Apply the trained YOLOv5x model to process each frame, detecting and recognizing pedestrians and vehicle targets within it. This enables us to accurately locate and label the positions of pedestrians and vehicles in the image, as shown in Figure 12.

**Figure 13:**
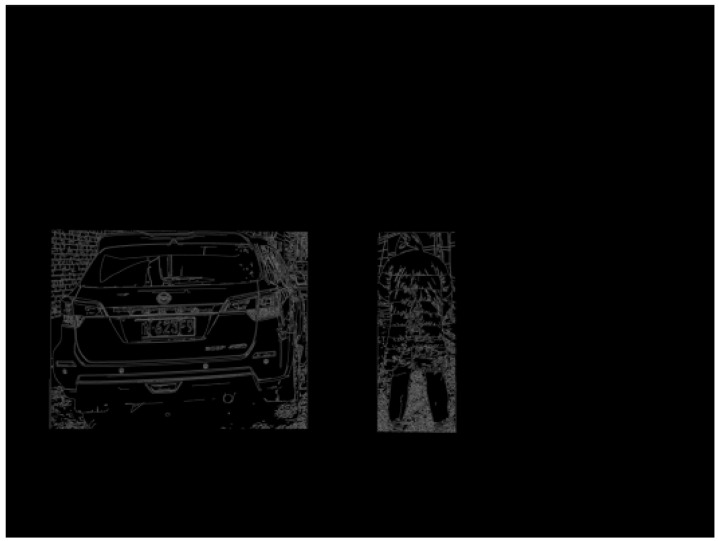
Edge Extraction Effect Image.

For each detected pedestrian and vehicle target, apply the Canny algorithm to extract their contours. This helps to more accurately determine the shapes and boundaries of pedestrians and vehicles. Determine the positions of the corner points to facilitate distance calculation later on, as shown in Figure 13.

By utilizing the known height of the camera and employing the same principle of pixel ratio within the same plane, the pixel distance between the right edge corner point of the car and the left edge corner point of the pedestrian was calculated. Then, the actual distance based on the formula was calculated. The measured actual distance was 85 cm, while the distance calculated by the algorithm was 84.1489 cm, resulting in a 1% error. The algorithm’s calculation was relatively accurate. Therefore, safety recommendations were proposed based on relevant traffic rules, and warnings were issued to pedestrians.

To further validate the accuracy and reliability of the algorithm, we conducted additional experiments. These experiments involved different scenarios, angles, and distances, and comparing the results with actual measurements.

First, we designed experiments to investigate the interaction between individual pedestrians and vehicles at different distances between the camera, pedestrians, and vehicles. The experimental setup was as follows:

(1) The camera was mounted on a streetlight at a height of 3 m.
(2) The pedestrian stood at three distances from the camera: 3 m, 7 m, and 14 m along the roadside.
(3) The lateral distance between the vehicle and the pedestrian was either 1.04 m or 2.20 m.

**Figure 14:**
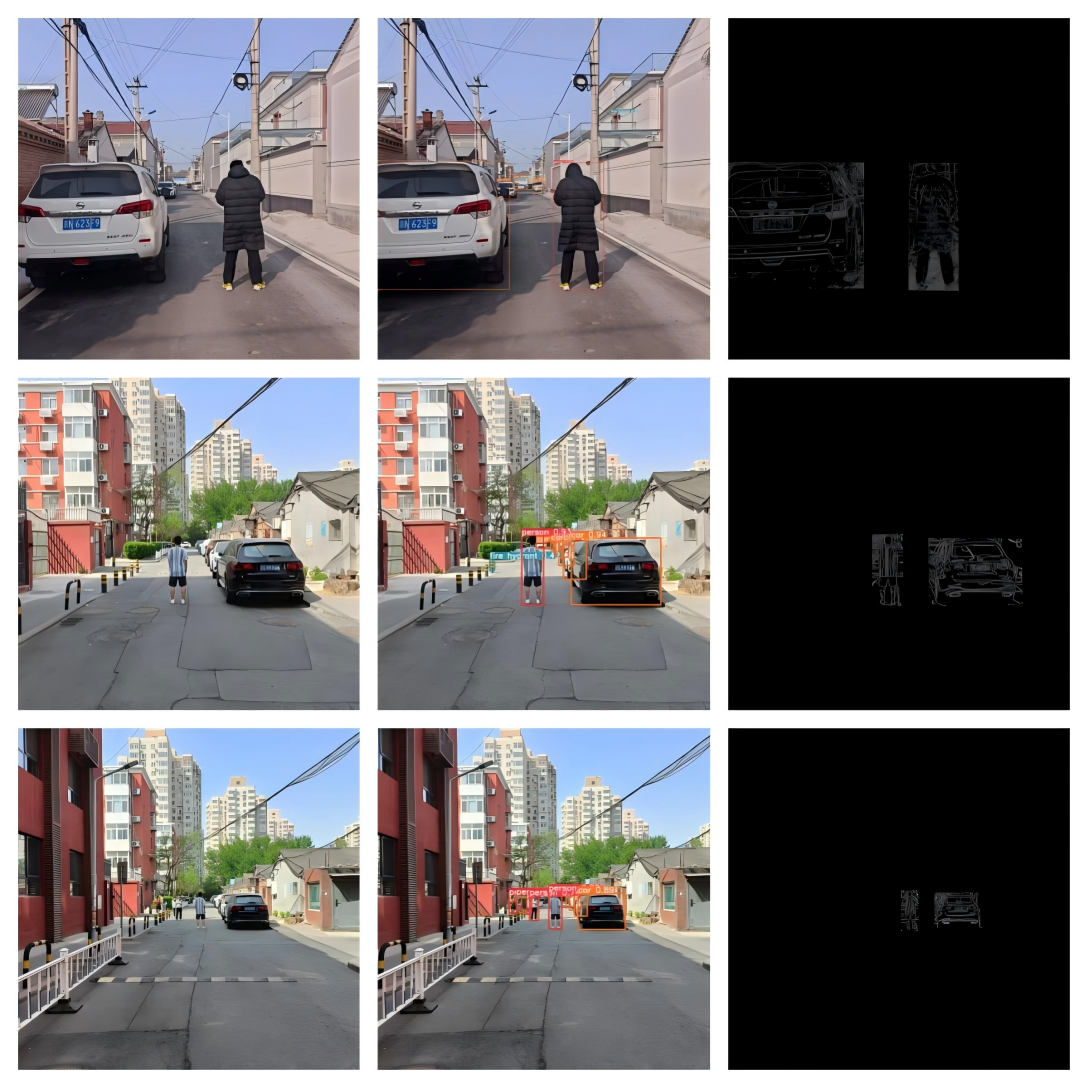
Schematic Diagram of Single Pedestrian System Effects.

The schematic diagram of the system is shown in Figure 14.

**Figure 15:**
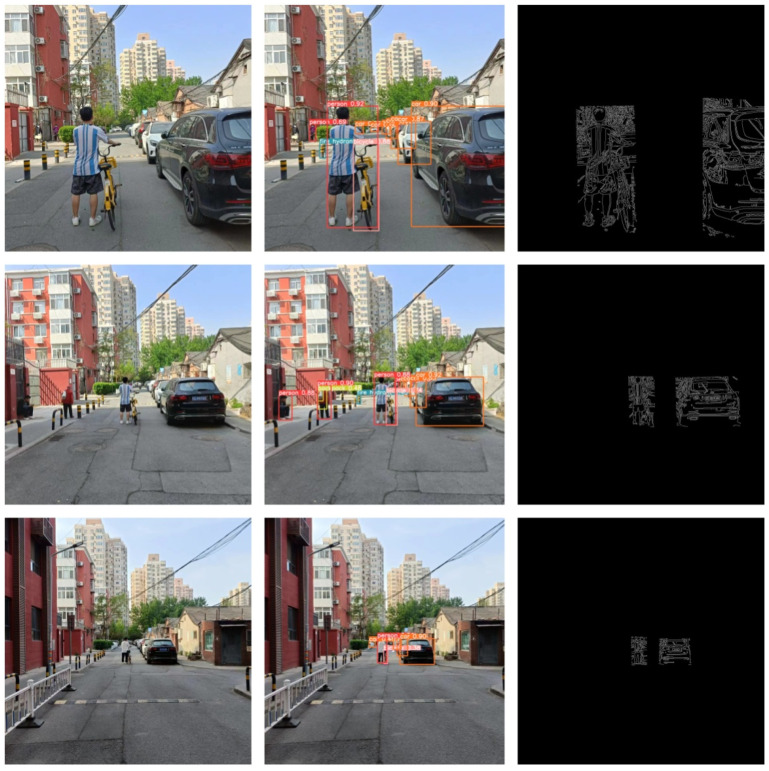
Schematic Diagram of Pedestrian Pushing Bicycle System Effects.

The experimental results indicated that the algorithm accurately calculated the distance between vehicles and pedestrians under different camera distances and pedestrian–vehicle distances. The specific data are shown in the Table 1:

Next, considering different pedestrian behaviors, we designed experiments to explore the relationship between pedestrians pushing bicycles and vehicles, with the experimental setup identical to the previous one. The schematic diagram of the system is shown in Figure 15.

**Figure 16:**
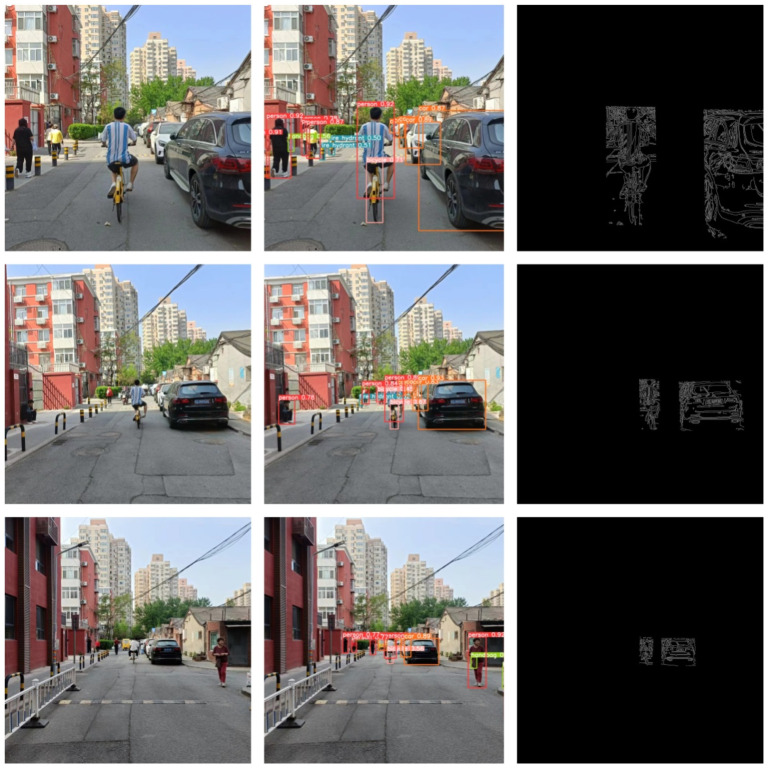
Schematic Diagram of Pedestrian Cycling System Effects.

The experimental results indicated that the algorithm could also accurately measure distances when pedestrians were pushing bicycles. The specific data are shown in the Table 2:

Next, considering the pedestrian cycling status, we designed experiments to explore the relationship between pedestrians riding bicycles and vehicles, with the experimental setup identical to the previous one. The schematic diagram of the system is shown in Figure 16.

**Figure 17:**
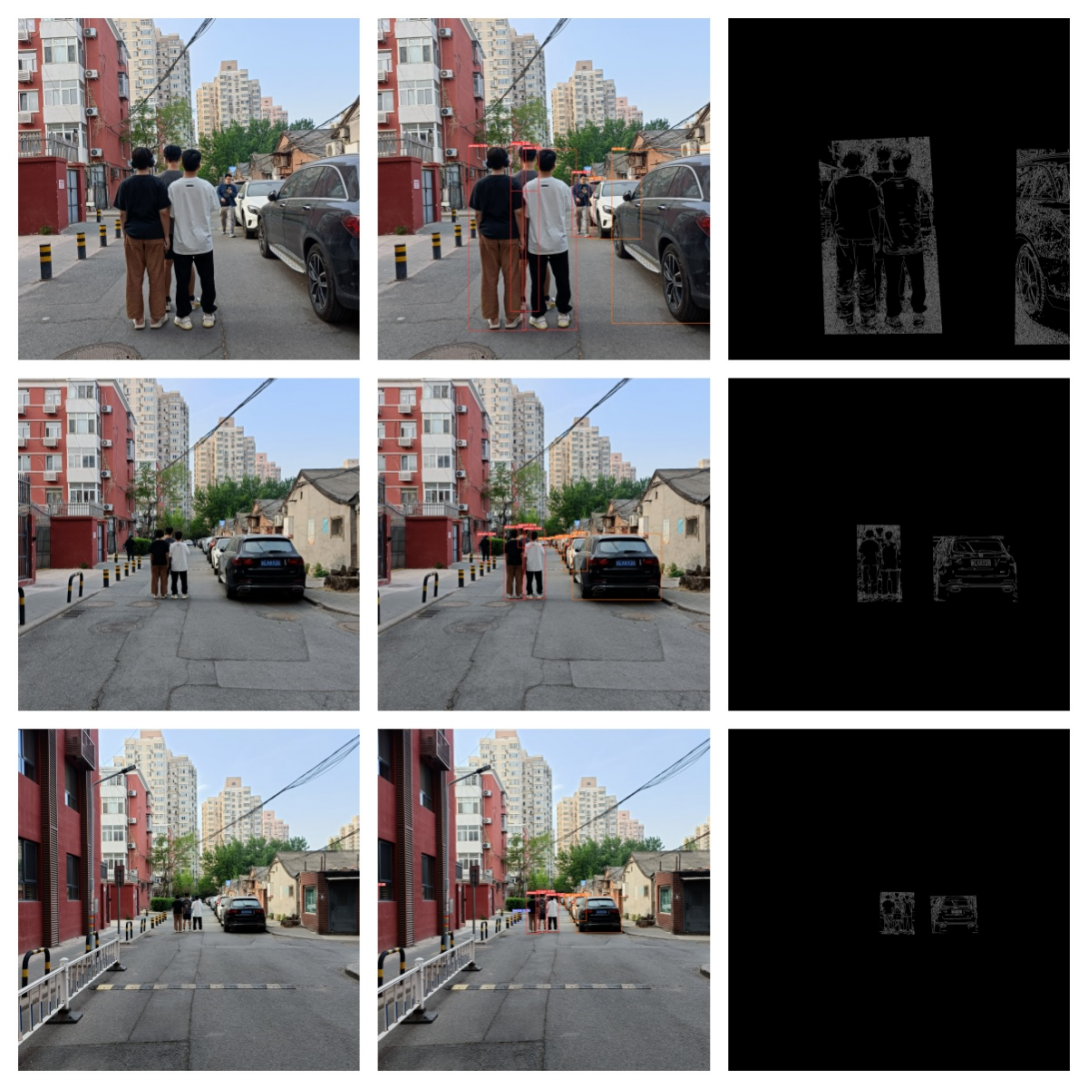
Schematic Diagram of Multiple Pedestrians System Effects.

The experimental results indicated that the algorithm could also measure distances accurately when pedestrians were riding bicycles. The specific data are shown in the Table 3:

Considering that pedestrians often cross roads together, we designed experiments to measure the distance between the nearest pedestrian and the vehicle when multiple pedestrians were walking together. The experimental setup was the same as before. The schematic diagram of the system is shown in Figure 17.

The experimental results indicated that the algorithm could also measure distances accurately when multiple pedestrians were walking together. The specific data are shown in the Table 4:

### 4.1. Validation of Overall System Model Accuracy

To verify the accuracy of the proposed vehicle distance detection and warning scheme, we compared it with the measurement data from the mid360 LiDAR. The mid360 LiDAR is a commonly used high-precision distance measurement device that can provide reliable ranging data. By comparing the measurement results of our system with those of the mid360 LiDAR under the same test conditions, we could effectively evaluate the accuracy and reliability of our method, as shown in Table 5:

The experimental results indicated that our method provided the most optimal distance estimation results across different ranges. Compared to the mid360 LiDAR, within a range of 10 m, the average error of our distance estimation method was reduced by up to 0.077 m. Even at distances beyond 10 m, the average error of our distance estimation results was guaranteed to be around 0.017 m. Compared to the mid360 LiDAR, the average error was significantly reduced, achieving the optimal result, and the accuracy of the system’s distance estimation was improved. Moreover, the maximum deviation of the average error across different ranges was about 0.018 m. Compared to the mid360 LiDAR, the deviation between the system’s estimation results at different distances was reduced, making the overall distance estimation system more stable and robust.

This demonstrates that the scheme has the potential to become an important tool for improving traffic safety, providing precise distance information for intelligent connected vehicles and autonomous driving vehicles, and ultimately contributing to the realization of holographic intersection vehicle–road collaborative driving.

### 4.2. Assessment of System’s Adaptability to Multiple Scenarios

In the experiments, only the system’s performance within residential areas was considered, neglecting its application in other scenarios. Therefore, the system was deployed in urban and rural streets for testing.

**Figure 18:**
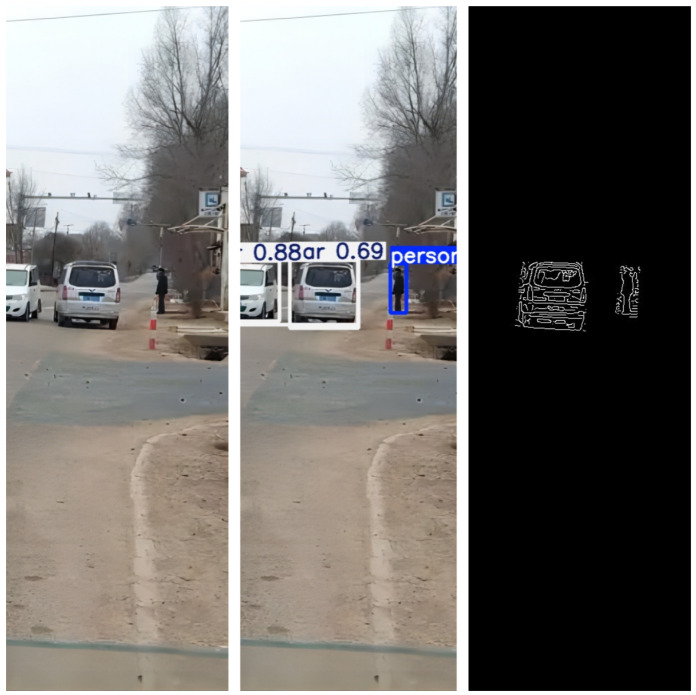
Application of the system on rural roads.

Rural roads typically have lower traffic density and wider road surfaces, but the road surfaces may be uneven, which may result in a larger field of view and uneven lighting conditions. The frequency of pedestrians, bicycles, and agricultural vehicles is relatively low, but occasional emergencies may occur, such as animals crossing the road [22], as shown in Figure 18.

To address these challenges and enhance the accuracy of the system, we integrated LiDAR as an auxiliary measurement tool in the system to better handle uneven road surfaces and emergencies [23]. This improved the system’s accuracy and its ability to respond to unexpected situations.

**Figure 19:**
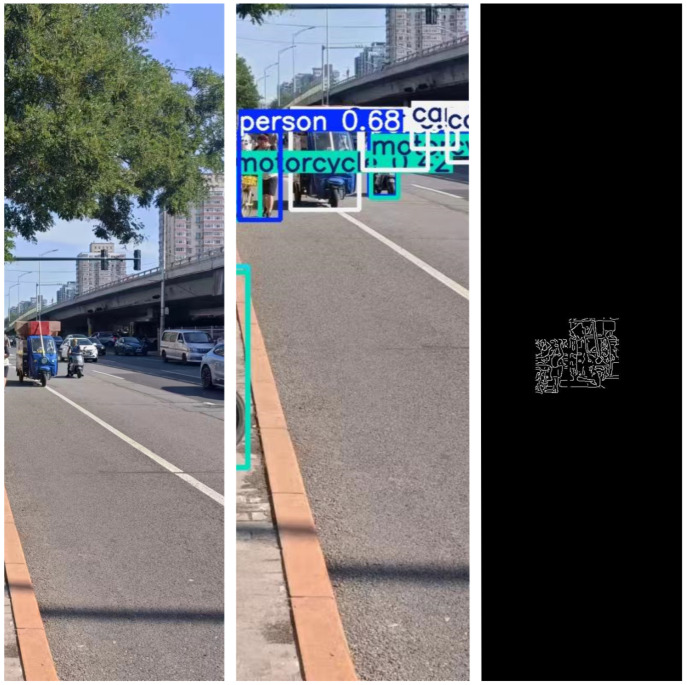
Application of the system on urban roads.

Urban roads have high traffic density and complex traffic environments, including various factors such as vehicles, pedestrians, bicycles, and traffic signals. There are significant variations in lighting conditions and road conditions, and congestion is often present, as shown in Figure 19.

We can see that urban roads are very busy, and the road conditions change frequently, so the system’s low-latency feature (overall latency controlled within 50 ms) ensures that safety warnings can be issued in real time in the busy urban road environment. In addition, by setting the camera’s sensitivity as a variable parameter and adjusting it in real time according to lighting changes, the system can handle data under different lighting conditions and maintain a high level of detection accuracy.

### 4.3. Evaluation of Vehicular Network Communication Performance

Implementing and deploying vehicular network architectures in real-world scenarios is challenging and costly. As a result, most research in the field of vehicular networks relies on simulation-based evaluations. In this study, we selected VanetMobiSim as the traffic simulator because it is open-source and has been validated against commercial simulators. VanetMobiSim supports an intelligent DriverModel with intersection management, generating realistic vehicle mobility models.

The experiments were conducted in two different speed environments to evaluate the system’s performance under varying speeds.

The experimental scenario design was as follows:

(1) Road environment selection:

Environment A: low-speed mixed traffic, with vehicle speeds below 30 km/h, and vehicles present at all intersections.

Environment B: medium-speed mixed traffic, with vehicle speeds between 30–60 km/h, and vehicles present at all intersections.

(2) Test vehicles and equipment:

Eight test vehicles were selected in each environment, each equipped with an onboard unit (OBU). Two Roadside Units (RSU) were installed at key points along the experimental route. During the experiment, IEEE 802.11p technology was used for data transmission between vehicles and RSUs.

**Figure 20:**
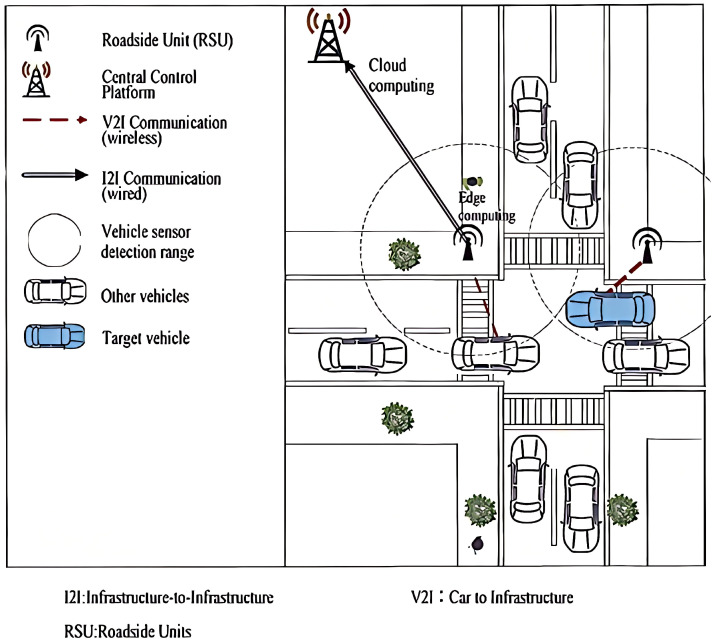
Experimental Scenario Diagram.

(3) Test scenario:

Vehicles traveled from one end of an intersection to the other, passing through the coverage areas of two RSUs. During this process, the RSUs transmitted safety warnings to the vehicles, as shown in Figure 20, to test the system’s seamless handover capability.

The experimental data are shown in Table 6.

The experimental data showed that the system maintained low latency across different environments and test scenarios, with an average latency of only 10–15 ms, ensuring real-time performance. The false positive rate and false negative rate were both below 2% and 1%, respectively, indicating the system’s accuracy and reliability in identifying potential hazards. The successful handover rate exceeded 97%, effectively achieving seamless transitions between RSUs and ensuring continuous communication. The safety warning accuracy rate was above 97%, allowing the system to provide precise safety alerts and significantly enhancing traffic safety.

### 4.4. Assessment of Overall System Computational Complexity

In this study, we proposed an automated vehicle distance detection and warning system based on roadside cameras and edge computing units, aimed at achieving vehicle–road collaboration in smart transportation. The core computational complexity analysis of the system is as follows:

Roadside cameras capture intersection videos in real time at 1080p resolution and 30 fps frame rate. Although the hardware performance has a relatively minor impact on the overall computational complexity of the system, it ensures the quality and continuity of data collection. The edge computing unit uses NVIDIA Jetson Xavier NX, which has powerful computing capabilities, capable of processing high-definition video streams in real time and running complex deep learning models. The YOLOv5x model, as the algorithm for object recognition, requires approximately 10 ms for each forward propagation, matching the frame rate of the camera, ensuring the real-time processing. The distance estimation algorithm based on single-image measurement and the principle of similar triangles has a computational complexity of O(1), capable of quickly estimating the distance in constant time.

Vehicular network communication uses IEEE 802.11p as the communication protocol, a wireless standard specifically designed for vehicular networks that provides low-latency and reliable communication in high-speed mobility environments. The protocol operates in the 5.9 GHz frequency band, supporting stable communication for vehicles traveling at high speeds. Through optimized transmission mechanisms, the protocol reduces communication latency in high-density traffic scenarios, ensuring efficient real-time data transmission.

The overall system latency, from video capture to the output of warning information, is controlled within 50 ms, meeting the real-time requirements. The hardware configuration of the edge computing unit, including a 6-core ARM CPU, 384-core Volta GPU, 8 GB of memory, and 512 GB of SSD storage, provides ample computing resources for the system. It not only supports the efficient operation of the YOLOv5x model but also meets other data processing and storage needs.

In summary, our system effectively controls computational complexity while maintaining high precision and real-time performance by optimizing algorithms and hardware configurations. This provides a solid technical foundation for achieving vehicle–road collaboration in holographic intersections and is expected to become an important tool in the field of traffic safety.

## 5. Safety Recommendations

According to relevant traffic regulations, when the distance between pedestrians and vehicles is less than 1 m, a series of measures must be taken to ensure the safety of both parties. Specific measures include:

1. Setting of warning lights and signals: The system automatically activates warning lights when the distance between pedestrians and vehicles is less than 1 m. These warning lights are usually located near roadsides or intersections and can attract the attention of pedestrians and drivers with a prominent flashing light. Warning signals include not only visual warning lights but also auditory alarms through an audio alarm system to enhance the effectiveness of the warning. The activation of warning lights and the issuance of warning sounds are triggered by the system’s internal real-time data processing and distance estimation algorithms.
2. Application of Vehicle-to-Everything (V2X) technology: When a hazardous distance is detected, the system immediately notifies nearby corresponding vehicles via vehicular network technology (V2X technology). This process involves transmitting alarm information to the vehicle’s onboard system. The driver of the vehicle receives a reminder to slow down and be informed to observe the movements of pedestrians. The onboard system may display relevant warning information and remind the driver to take appropriate action through sound or visual cues [24].
3. Pedestrian safety reminders: The system also issues warnings to pedestrians through public display screens or smartphone applications, reminding them to pay attention to their surroundings. Pedestrians should be aware of these warning signals and maintain a safe distance from vehicles to ensure their own safety. These reminders include warning signs, dynamic message boards, or mobile notifications, with the aim of enhancing pedestrians’ alertness.

Implementation methods:

1. Implementation of warning lights and audio alarm systems: The cameras in the system capture real-time distance data between pedestrians and vehicles. Once the distance is detected to be less than 1 m, the edge computing unit quickly processes these data and triggers the warning lights and audio alarm system through control signals. These systems can be connected to existing traffic infrastructure to ensure timely and effective warnings [25].
2. Implementation of V2X technology: The V2X system sends alarm information to vehicles through wireless communication technology (IEEE 802.11p protocol). The onboard computing system of the vehicle receives this information and displays warning information on the driver’s display screen, while also issuing an audible reminder. This requires effective integration of the system with the vehicle’s onboard communication unit to achieve real-time data transmission and processing.
3. Implementation of pedestrian safety reminders: Pedestrian warning information is published through public display screens, smartphone applications, or road information boards. The system connects to the city’s public information system interface to convey warning information to pedestrians. These reminder systems can be updated in real time via wireless networks or wired connections to ensure that pedestrians receive timely safety prompts [26].

By implementing these measures, the system can effectively enhance traffic safety, reduce potential accident risks, ensure a safe distance between pedestrians and vehicles, and safeguard the safety of pedestrians and drivers.

## 6. Conclusions

This study proposed a traffic safety warning system that integrated YOLOv5x, monocular distance measurement, and Vehicle-to-Everything (V2X) technology. The system provides an effective solution for traffic safety by identifying targets and estimating distances. Utilizing V2X technology, the system can notify drivers to make corresponding adjustments and warn pedestrians of changes in their surroundings through warning lights. Experimental results showed that the system could accurately identify vehicles and pedestrians and precisely measure the distance between targets using the method of similar triangles. Based on distance estimation, the system generates corresponding safety recommendations, providing better traffic safety protection for pedestrians. The system is expected to be applied in real traffic environments, reducing the occurrence of traffic accidents and ensuring pedestrian safety.

Outlook:

In the future, with the continuous advancement of technology and the development of intelligent transportation systems, the traffic safety warning system proposed in this paper is expected to achieve optimization and expansion in the following aspects:

1. System performance improvement: The performance of the YOLOv5x model can be further optimized to improve the accuracy and processing speed of target recognition. At the same time, by improving the monocular measurement algorithm, the accuracy of distance estimation can be further enhanced, enabling the system to work stably in various complex environments.
2. Adaptability to multiple scenarios: The system can be extended to more traffic scenarios, such as highways and complex intersections. By conducting adaptability tests in different scenarios, the system’s functions can be perfected to provide effective safety protection in a wider range of traffic environments.
3. Real-time data analysis: We can combine more sensor data and real-time data analysis technology to enhance the system’s dynamic response capabilities to traffic flow, traffic signs, weather, and other factors, to provide more accurate traffic safety recommendations.
4. Intelligence and adaptive features: We can introduce artificial intelligence technology to enhance the system’s adaptive capabilities, enabling it to automatically adjust warning strategies and early warning mechanisms according to real-time traffic conditions, further improving traffic safety.
5. User experience and system integration: We can optimize the user interface and user experience, making it more convenient for drivers and pedestrians to obtain and understand the safety information provided by the system. At the same time, we can explore integration with other intelligent transportation systems to form a more comprehensive traffic safety solution.

Through these improvements and expansions, the system will be better able to meet the safety needs of future traffic environments, providing strong support for the development of intelligent connected vehicles and autonomous driving technology.

## Figures and Tables

**Table 1:** Distancemeasurement data table for a single pedestrian.

	Quantity	1	2	3	4	5	6
Distance	Distance between camera and pedestrian (m)	3	3	7	7	14	14
Verification	Actual distance between pedestrian and vehicle (m)	1.04	2.20	1.04	2.20	1.04	2.20
(Single pedestrian)	Distance between pedestrian and vehicle measured by the system (m)	1.051	2.228	1.031	2.213	1.023	2.228
	Absolute error (m)	0.011	0.028	0.009	0.013	0.017	0.028

**Table 2:** Distance measurement data table for a pedestrian pushing a bicycle.

	Quantity	1	2	3	4	5	6
Distance	Distance between camera and pedestrian (m)	3	3	7	7	14	14
Verification	Actual distance between pedestrian and vehicle (m)	1.04	2.20	1.04	2.20	1.04	2.20
(Pedestrian pushing a bicycle)	Distance between pedestrian and vehicle measured by the system (m)	1.036	2.223	1.065	2.212	1.041	2.216
	Absolute error (m)	0.004	0.023	0.025	0.012	0.001	0.016

**Table 3:** Distance measurement data table for a pedestrian cycling.

	Quantity	1	2	3	4	5	6
Distance	Distance between camera and pedestrian (m)	3	3	7	7	14	14
Verification	Actual distance between pedestrian and vehicle (m)	1.04	2.20	1.04	2.20	1.04	2.20
(Pedestrian cycling)	Distance between pedestrian and vehicle measured by the system (m)	1.058	2.213	1.015	2.229	1.032	2.229
	Absolute error (m)	0.018	0.013	0.025	0.029	0.008	0.029

**Table 4:** Distance measurement data table for multiple pedestrians.

	Quantity	1	2	3	4	5	6
Distance	Distance between camera and pedestrian (meters)	3	3	7	7	14	14
Verification	Actual distance between pedestrian and vehicle (meters)	1.04	2.20	1.04	2.20	1.04	2.20
(Multiple pedestrians)	distance between pedestrian and vehicle measured by the system (meters)	1.054	2.221	1.045	2.229	1.036	2.229
	Absolute error (meters)	0.014	0.021	0.005	0.029	0.004	0.029

**Table 5:** Error comparison.

Distance	Average Error of Proposed Method (m)	Average Error of mid360 LiDAR (m)
3 m	0.016	0.021
7 m	0.018	0.096
14 m	0.016	0.214

**Table 6:** Vehicular network performance evaluation.

Environment	Average Latency (ms)	False Positive Rate (%)	False Negative Rate (%)	Successful Handover Rate (%)	Safety Warning Accuracy (%)
A	15	1.2	0.5	99.0	98.5
B	12	1.0	0.8	97.5	98.0

## Data Availability

Data are contained within the article.
